# Efficacy of interventions with live combined *Bacillus subtilis* and *Enterococcus faecium* enteric-coated capsules in metabolic associated fatty liver disease patients: a meta-analysis of randomized controlled trials

**DOI:** 10.3389/fphar.2025.1610426

**Published:** 2025-05-27

**Authors:** Yutong Wu, Hao Wu, Hang Yi

**Affiliations:** ^1^ Clinical Medicine, Chongqing Medical University, University of Leicester Joint Institute, Chongqing, China; ^2^ Department of Gastroenterology, West China Hospital, Sichuan University, Chengdu, China; ^3^ Department of Gastroenterology, Second Affiliated Hospital of Chongqing Medical University, Chongqing, China

**Keywords:** metabolic associated fatty liver disease, live combined *Bacillus subtilis* and *Enterococcus* faecium enteric-coated capsules, liver function, metabolic status, inflammation

## Abstract

**Objective:**

Metabolic associated fatty liver disease (MAFLD) is a common liver disease worldwide. However, effective therapies are still lacking. This meta-analysis aimed to compare the efficacy of interventions with or without live combined *Bacillus subtilis* and *Enterococcus faecium* (LCBE) enteric-coated capsules in MAFLD patients, thereby providing some reference for clinicians in optimizing treatment strategies.

**Methods:**

Embase, PubMed, Web of Science, Cochrane Library, Wan Fang, China Science and Technology Journal Database, China National Knowledge Infrastructure, and China Biomedical Literature Service System were searched for relevant randomized controlled trials (RCTs). MAFLD patients receiving interventions with or without LCBE enteric-coated capsules were categorized into the experimental or control group, respectively.

**Results:**

This meta-analysis included 21 RCTs with 1783 MAFLD patients. The effective rate was higher in the experimental group than in the control group (*P* < 0.001). The normal and light fatty liver rate was increased in the experimental group compared to the control group (*P* = 0.003). Aspartate transaminase, alanine aminotransferase, and gamma-glutamyl transferase were lower in the experimental group than the control group (all *P* < 0.01). Body mass index, fasting blood glucose, triglyceride, total cholesterol, and low-density lipoprotein cholesterol were lower, and high-density lipoprotein cholesterol was higher in the experimental group than the control group (all *P* < 0.05). C-reactive protein, interleukin-6, tumor necrosis factor-alpha, and endotoxin were lower in the experimental group than in the control group (all *P* < 0.01).

**Conclusion:**

Interventions containing LCBE enteric-coated capsules exhibit satisfactory efficacy, which improve liver function, metabolic status, and inflammation compared to those without LCBE enteric-coated capsules in MAFLD patients.

## 1 Introduction

Metabolic associated fatty liver disease (MAFLD), characterized by excessive hepatic lipid accumulation, affects over 30% of the global population (Han et al., 2023; Teng et al., 2023). While early-stage MAFLD is usually harmless, it can progress to severe liver damage, including cirrhosis and hepatocellular carcinoma (Grander et al., 2023; Leow et al., 2023). Moreover, persistent hepatic steatosis increases the risks of cardiovascular diseases and type 2 diabetes, which are the leading causes of poor clinical outcomes in MAFLD patients (Powell et al., 2021). The gut-liver axis plays a pivotal role in MAFLD progression (Hsu and Schnabl, 2023). Specifically, gut-derived microbial products (such as lipopolysaccharide) directly influence hepatic inflammation and lipid metabolism through portal circulation, while liver-derived metabolites conversely shape gut microbiota composition (Hsu and Schnabl, 2023; Tilg et al., 2022). This bidirectional communication creates a pathological feedback loop that exacerbates liver injury, which is manifested as elevated liver enzymes, metabolic dysfunction, aggravated inflammation, and impaired liver function (Hsu and Schnabl, 2023). Therefore, interventions that regulate gut dysbiosis, such as probiotic treatment, have emerged as a promising strategy for treating MAFLD patients (Fang et al., 2022).

Currently, several probiotics, such as *Lactobacillus*, are available in commercial markets, and some previous meta-analyses have explored the efficacy of these probiotics for the treatment of MAFLD (Li et al., 2022; Carpi et al., 2022; Rong et al., 2023; Yang et al., 2021). For instance, a previous meta-analysis reported that probiotics-containing interventions improved energy metabolism biomarkers compared to interventions without probiotics in MAFLD patients (Li et al., 2022). Another previous meta-analysis indicated that probiotics-containing interventions improved liver function and reduced blood lipid levels compared to interventions without probiotics in MAFLD patients (Yang et al., 2021).

Live combined *Bacillus subtilis* and *Enterococcus faecium* (LCBE) enteric-coated capsule is a probiotic preparation that consists of two probiotic bacteria, *E. faecium* R-026 and *Bacillus subtilis* R-179, at a ratio of 9:1 (Sohail et al., 2018). An *in vivo* experiment found that LCBE treatment improves liver function, lipid profiles, and inflammation to attenuate MAFLD progression in mice (Jiang et al., 2021). Additionally, some clinical studies have also explored the efficacy of LCBE enteric-coated capsules in MAFLD patients (Yang et al., 2012; Zhao et al., 2013; Luo et al., 2014; Hu et al., 2015; Yang, 2015; Yi and Zeng, 2015; Mei et al., 2016; He et al., 2017; Zhan et al., 2017; Zhao, 2017; Liu et al., 2018; Wang, 2018; Wang et al., 2018; Deng, 2020; Sun et al., 2020; Xu, 2020; Zhang, 2021; Xue et al., 2022; Li et al., 2023; Wang and Dai, 2023; Liu et al., 2024). However, the sample sizes of most studies are relatively small, which limits the statistical power. On the other hand, inconsistent findings exist among previous studies. Therefore, to provide valuable insights for clinical practice, a pooled analysis is required to synthesize data from these studies and evaluate the overall efficacy of LCBE enteric-coated capsules in MAFLD patients.

Accordingly, the current meta-analysis aimed to compare the efficacy of interventions with or without LCBE enteric-coated capsules in MAFLD patients.

## 2 Methods

### 2.1 Search strategy

Multiple electronic databases, including Embase, PubMed, Web of Science, Cochrane Library, Wan Fang, China Science and Technology Journal Database (VIP), China National Knowledge Infrastructure (CNKI), and China Biomedical Literature Service System (SinoMed) were searched comprehensively using the following key terms: “*Bacillus Subtilis* and *Enterococcus Faecium*”, “live combined *Bacillus Subtilis* and *Enterococcus Faecium* Enteric-coated Capsules”, “Medilac-S”, “non-alcoholic fatty liver disease”, “NAFLD”, “non-alcoholic steatohepatitis”, “MAFLD”, “metabolic associated fatty liver disease”, and “NASH”. The search was restricted to studies published before 2 December 2024. Additionally, we manually reviewed the references of selected studies to identify potentially relevant articles.

### 2.2 Inclusion and exclusion criteria

This meta-analysis was conducted according to populations, interventions, comparators, outcomes, and study designs (PICOS) criteria. The inclusion criteria for eligible studies were: 1) Population (P): patients diagnosed with MAFLD; 2) Interventions (I): patients in the experimental group receiving interventions with LCBE enteric-coated capsules; 3) Comparators (C): patients in the control groups receiving interventions without LCBE enteric-coated capsules; 4) Outcomes (O): efficacy-related results; 5) Study designs (S): randomized controlled trails (RCTs) published in English or Chinese. The exclusion criteria for eligible studies were: 1) reviews, meta-analyses, animal research, or case reports; 2) studies conducted by the same authors and with repeated patients and assessments. The qualities of the final included studies were evaluated by the Risk Of Bias (ROB) 2.0 tool (Sterne et al., 2019).

### 2.3 Data extraction

The data extracted included details such as the first author, year of publication, sample size, demographics, and intervention. Moreover, the evaluation indicators related to the efficacy of LCBE enteric-coated capsules were screened for system analysis. In this meta-analysis, the efficacy-related indicators involved the improvement of MAFLD (effective rate, and normal and light fatty liver rate), liver function parameters [aspartate transaminase (AST), alanine aminotransferase (ALT), and gamma-glutamyl transferase (GGT)], metabolic parameters [body mass index (BMI), fasting blood glucose (FBG), triglyceride (TG), total cholesterol (TC), high-density lipoprotein cholesterol (HDL-C), and low-density lipoprotein cholesterol (LDL-C)], and inflammation markers [C-reactive protein (CRP), interleukin (IL)-6, tumor necrosis factor-alpha (TNF-α), and endotoxin]. For the effective rate, the criteria involved in the studies were not uniform, including liver function, blood lipid level, fatty liver improvement, and other aspects, but the efficacy results were divided into three categories: markedly effective, effective, and ineffective. To include as many studies as possible for pooled analysis, this meta-analysis combined the effective rates from different definitions.

### 2.4 Effect size and models

The effect sizes were pooled using the odds ratio (OR) with a 95% confidence interval (CI) or the standard (std.) mean difference (SMD) with a 95% CI. The reason for choosing SMD was due to the inconsistency in units of continuous variables. The former was used to pool the ‘effective rate’ and ‘normal and mild fatty liver rate’; while the latter was used to pool the remaining continuous variables. A random-effects or fixed-effects model was selected according to the I^2^ test results. When I^2^ exceeded 50%, indicating significant heterogeneity, the random-effects model was utilized; otherwise, the fixed-effects model was applied.

### 2.5 Statistics

Publication bias was assessed via Begg’s test. If the *P* value of Begg’s test was less than 0.05, it indicated that there might be significant publication bias. In this case, the trim-and-filling method was used to adjust the publication bias, and the effect size was recalculated to obtain a more reliable estimate. Sensitivity analyses were conducted by excluding one by one to check the robustness of the overall findings. R software (version 4.4.2) was applied for data analyses. A *P* value <0.05 indicated significance.

## 3 Results

### 3.1 Study screen procedure

**FIGURE 1:**
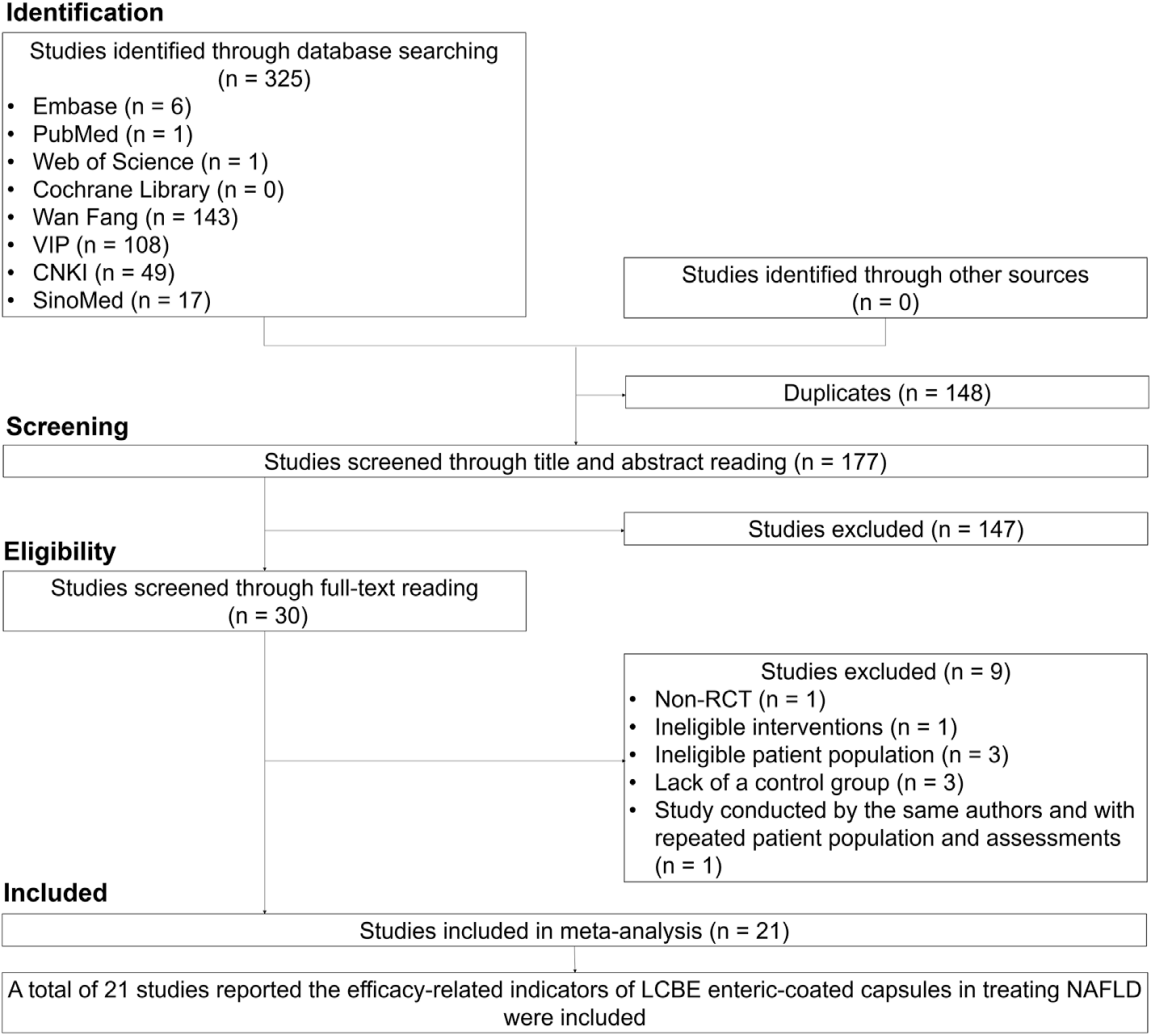
Study screen procedure.

A total of 325 studies were identified through database searching, and 148 duplicates were excluded. Then, 177 studies were screened through title and abstract reading, and 147 studies were excluded. Subsequently, 30 studies were screened through full-text reading, and 9 studies were excluded. Twenty-one studies that reported the efficacy of LCBE enteric-coated capsules in MAFLD patients were finally included (Yang et al., 2012; Zhao et al., 2013; Luo et al., 2014; Hu et al., 2015; Yang, 2015; Yi and Zeng, 2015; Mei et al., 2016; He et al., 2017; Zhan et al., 2017; Zhao, 2017; Liu et al., 2018; Wang, 2018; Wang et al., 2018; Deng, 2020; Sun et al., 2020; Xu, 2020; Zhang, 2021; Xue et al., 2022; Li et al., 2023; Wang and Dai, 2023; Liu et al., 2024; Figure 1).

### 3.2 Characteristics of included studies

This meta-analysis included 1783 MAFLD patients. Patients who received interventions with LCBE enteric-coated capsules were assigned to the experimental group (N = 891), while patients who received interventions without LCBE enteric-coated capsules were assigned to the control group (N = 892). The mean age of patients in the experimental group ranged from 35.4 to 62.1 years, which was 34.8 to 63.0 years in the control group. Four studies did not provide information on age. The information on sample size, age, sex, interventions, and outcomes of each study is shown in Table 1.

**TABLE 1:** Features of included studies.

Study ID	Sample size, n		Age (years), mean ± SD		Male/Female, n		Intervention		Evaluation indicators
	Experimental	Control	Exp erimental	Control	Experimental	Control	Experimental	Control	
Yang et al. (2012)	30	30	NA	NA	NA	NA	LCBE enteric-coated capsules + Diamine glycyrrhizinate enteric-coated capsules	Diamine glycyrrhizinate enteric-coated capsules	ALT, Endotoxin, TNF-α, IL-6
Zhao et al. (2013)	30	30	43.6 ± 12.4	45.7 ± 7.3	22/8	24/6	LCBE enteric-coated capsules + Atomolan + Polyene phosphatidylcholine capsule	Atomolan + Polyene phosphatidylcholine capsule	ALT, TNF-α
Luo et al. (2014)	41	44	41.8 ± 10.8	42.6 ± 6.2	29/12	32/12	LCBE enteric-coated capsules + Bicyclol tablet	Bicyclol tablet	AST, ALT, GGT, TG, TC, TNF-α
Hu et al. (2015)	53	54	40.0 ± 12.2	39.6 ± 11.0	30/23	35/19	LCBE enteric-coated capsules + Xuezhikang capsule + Glucurolactone	Xuezhikang capsule + Glucurolactone	Effective rate, BMI, AST, ALT, TC
Yang (2015)	39	39	42.1 ± 4.6	41.9 ± 4.4	25/14	23/16	LCBE enteric-coated capsules + Diamine glycyrrhizinate enteric-coated capsules	Diamine glycyrrhizinate enteric-coated capsules	ALT, GGT, TG, TC, HDL-C, LDL-C, Endotoxin
Yi and Zeng (2015)	40	40	47.6 ± 4.5	46.7 ± 3.7	30/10	31/9	LCBE enteric-coated capsules + Atomolan + Polyene phosphatidylcholine capsule	Atomolan + Polyene phosphatidylcholine capsule	AST, ALT, GGT, TNF-α
Mei et al. (2016)	44	44	57.3 ± 5.8	54.6 ± 5.2	21/23	19/25	LCBE enteric-coated capsules + Basic treatment	Basic treatment	Effective rate, ALT, TG, Endotoxin, TNF-α
He et al. (2017)	35	35	62.1 ± 10.4	63.0 ± 10.7	18/17	20/15	LCBE enteric-coated capsules + Polyene phosphatidylcholine capsule	Polyene phosphatidylcholine capsule	BMI, AST, ALT, TG, TC
Zhan et al. (2017)	37	35	NA	NA	NA	NA	LCBE enteric-coated capsules + Polyene phosphatidylcholine capsule + Vitamin E soft capsules	Polyene phosphatidylcholine capsule + Vitamin E soft capsules	Normal and light fatty liver rate, FBG, AST, ALT, GGT, TG, TC, HDL-C, LDL-C, Endotoxin, CRP, TNF-α, IL-6
Zhao (2017)	60	60	NA	NA	NA	NA	LCBE enteric-coated capsules + Fufang Danshen injection	Fufang Danshen injection	AST, ALT, TG, TC, HDL-C, LDL-C
Liu et al. (2018)	39	39	NA	NA	NA	NA	LCBE enteric-coated capsules + Polyene phosphatidylcholine capsule	Polyene phosphatidylcholine capsule	Normal and light fatty liver rate, TG, TC, HDL-C, LDL-C
Wang et al. (2018)	59	59	50.7 ± 7.8	50.2 ± 7.7	40/19	41/18	LCBE enteric-coated capsules + Polyene phosphatidylcholine capsule	Polyene phosphatidylcholine capsule	Effective rate, AST, ALT, GGT, TG, TC, HDL-C, LDL-C, TNF-α, IL-6
Wang (2018)	50	50	42.2 ± 5.8	44.3 ± 4.7	35/15	34/16	LCBE enteric-coated capsules + Polyene phosphatidylcholine capsule	Polyene phosphatidylcholine capsule	FBG, AST, ALT, TG, TC, HDL-C, LDL-C, TNF-α
Deng (2020)	47	47	49.4 ± 4.6	50.0 ± 4.6	35/12	33/14	LCBE enteric-coated capsules + Polyene phosphatidylcholine capsule	Polyene phosphatidylcholine capsule	Effective rate, AST, ALT, TG, TC
Sun et al. (2020)	52	52	43.8 ± 7.9	44.1 ± 8.2	27/25	26/26	LCBE enteric-coated capsules + Polyene phosphatidylcholine capsule	Polyene phosphatidylcholine capsule	FBG, AST, ALT, TG, TC, HDL-C, LDL-C
Xu (2020)	42	42	41.3 ± 4.6	41.4 ± 4.6	28/14	27/15	LCBE enteric-coated capsules + Polyene phosphatidylcholine capsule + Bicyclol tablet	Polyene phosphatidylcholine capsule + Bicyclol tablet	Effective rate, BMI, AST, ALT
Zhang (2021)	30	30	41.7 ± 3.3	41.2 ± 3.5	16/14	17/13	LCBE enteric-coated capsules + Bifid Tiple Viable	Bifid Tiple Viable	FBG, AST, ALT, GGT, Endotoxin, CRP, TNF-α
Xue et al. (2022)	49	49	41.3 ± 7.5	41.8 ± 6.2	30/19	29/20	LCBE enteric-coated capsules + Exenatide	Exenatide	ALT, GGT, TG, TC, HDL-C, LDL-C, Endotoxin
Li et al. (2023)	46	47	40.1 ± 13.9	40.4 ± 13.9	27/19	32/15	LCBE enteric-coated capsules + Bicyclol tablet	Bicyclol tablet	Effective rate, Normal and light fatty liver rate, BMI, AST, ALT, GGT, TG, TC, IL-6
Wang and Dai (2023)	30	30	45.8 ± 6.9	45.0 ± 7.6	16/14	17/13	LCBE enteric-coated capsules + Bicyclol tablet	Bicyclol tablet	AST, ALT, TG, TC, HDL-C, LDL-C, CRP, IL-6
Liu et al. (2024)	38	36	35.4 ± 6.1	34.8 ± 5.9	22/16	22/14	LCBE enteric-coated capsules + Lifestyle modification	Lifestyle modification	Effective rate, BMI, FBG, AST, ALT, TG, TC, LDL-C, Endotoxin, TNF-α

SD, standard deviation; NA, not available; LCBE, live combined *bacillus subtilis* and *enterococcus* faecium; ALT, alanine aminotransferase; TNF-α, tumor necrosis factor-alpha; IL-6, interleukin-6; AST, aspartate transaminase; GGT, gamma-glutamyl transferase; TG, triglyceride; TC, total cholesterol; BMI, body mass index; HDL-C, high-density lipoprotein cholesterol; LDL-C, low-density lipoprotein cholesterol; FBG, fasting blood glucose; CRP, C-reactive protein.

### 3.3 Quality assessment

Most studies were assessed as having low risk or unclear risk for 5 domains. However, Hu et al. (2015) and Deng (2020) were assessed as having high risk for bias in the measurement of the outcome. Regarding overall risk of bias, 12 studies were assessed as having low risk, 7 studies were assessed as having unclear risk, and 2 studies were assessed as having high risk (Table 2).

**TABLE 2:** Risk of bias via Cochrane ROB tool 2.0.

Study ID	Domain 1	Domain 2	Domain 3	Domain 4	Domain 5	Overall
Yang et al. (2012)	Unclear	Low risk	Low risk	Low risk	Low risk	Unclear
Zhao et al. (2013)	Low risk	Low risk	Low risk	Low risk	Low risk	Low risk
Luo et al. (2014)	Low risk	Low risk	Low risk	Unclear	Low risk	Unclear
Hu et al. (2015)	Unclear	Low risk	Unclear	High risk	Low risk	High risk
Yang (2015)	Low risk	Low risk	Low risk	Low risk	Low risk	Low risk
Yi and Zeng (2015)	Low risk	Low risk	Unclear	Unclear	Low risk	Unclear
Mei et al. (2016)	Low risk	Low risk	Unclear	Low risk	Low risk	Unclear
He et al. (2017)	Low risk	Low risk	Low risk	Low risk	Low risk	Low risk
Zhan et al. (2017)	Low risk	Low risk	Low risk	Low risk	Low risk	Low risk
Zhao (2017)	Low risk	Low risk	Low risk	Unclear	Low risk	Unclear
Liu et al. (2018)	Low risk	Low risk	Low risk	Low risk	Low risk	Low risk
Wang et al. (2018)	Low risk	Low risk	Low risk	Low risk	Low risk	Low risk
Wang (2018)	Low risk	Low risk	Low risk	Low risk	Low risk	Low risk
Deng (2020)	Low risk	Low risk	Unclear	High risk	Low risk	High risk
Sun et al. (2020)	Low risk	Low risk	Low risk	Low risk	Low risk	Low risk
Xu (2020)	Low risk	Low risk	Low risk	Low risk	Low risk	Low risk
Zhang (2021)	Low risk	Low risk	Low risk	Low risk	Low risk	Low risk
Xue et al. (2022)	Low risk	Low risk	Unclear	Low risk	Low risk	Unclear
Li et al. (2023)	Low risk	Low risk	Unclear	Low risk	Low risk	Unclear
Wang and Dai (2023)	Low risk	Low risk	Low risk	Low risk	Low risk	Low risk
Liu et al. (2024)	Low risk	Low risk	Low risk	Low risk	Low risk	Low risk

Domain 1: bias arising from the randomization process.

Domain 2: bias due to deviations from intended interventions.

Domain 3: bias due to missing outcome data.

Domain 4: bias in measurement of the outcome.

Domain 5: bias in selection of the reported result.

### 3.4 Comparison of MAFLD improvement between experimental and control groups

**FIGURE 2:**
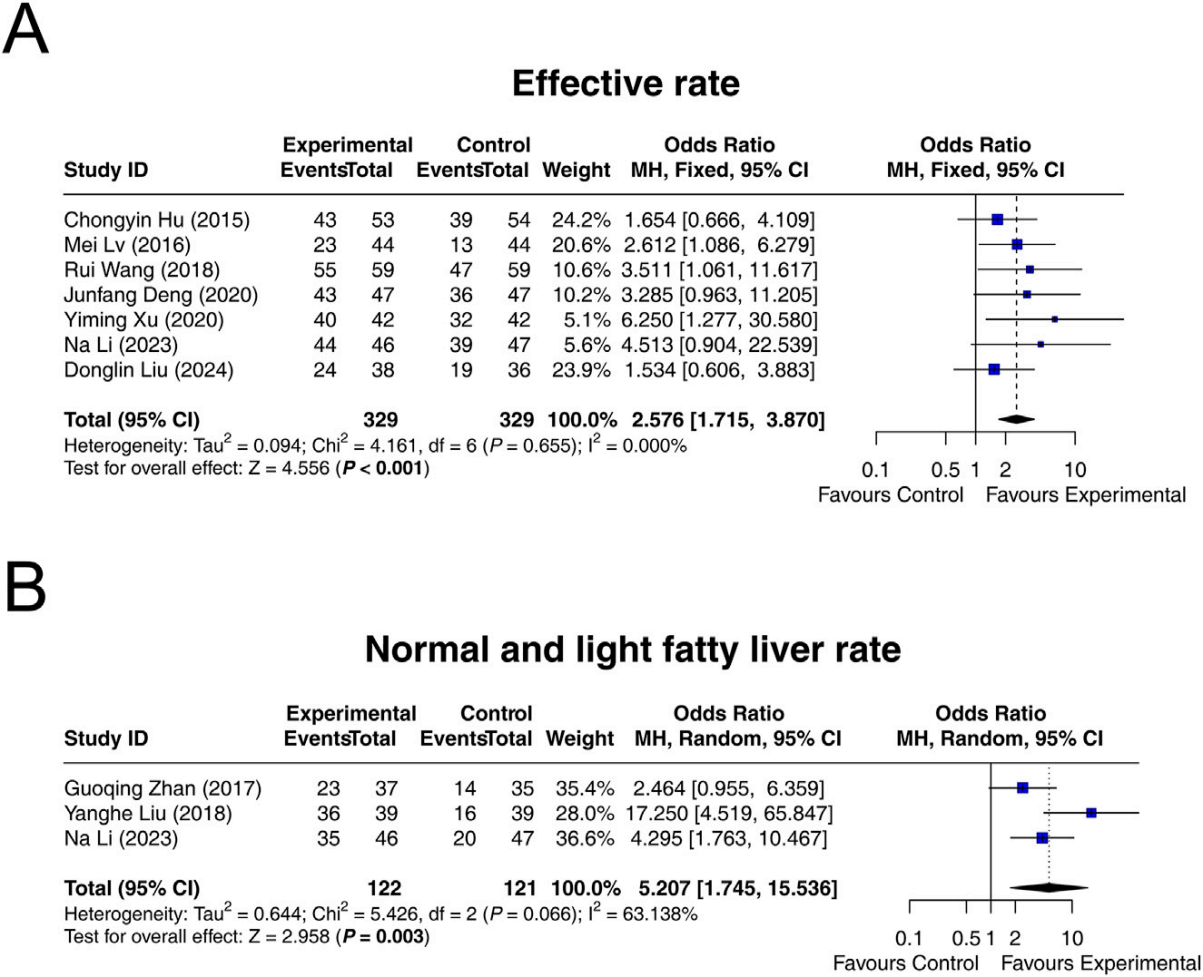
Forest plots of MAFLD improvement. Effective rate **(A)**, as well as normal and light fatty liver rate **(B)**, in the control and experimental groups.

Seven studies reported the effective rate, and heterogeneity did not exist among these studies (I^2^ = 0.000%, *P* = 0.655). Fixed-effects model suggested that interventions containing LCBE enteric-coated capsules increased the effective rate compared to interventions without LCBE enteric-coated capsules (OR = 2.576; 95% CI: 1.715, 3.870; *P* < 0.001) (Figure 2A).

Three studies reported normal and light fatty liver rate after treatment. Heterogeneity was found among these studies (I^2^ = 63.138%, *P* = 0.066). Random-effects model indicated that interventions containing LCBE enteric-coated capsules increased the normal and light fatty liver rate compared to interventions without LCBE enteric-coated capsules (OR = 5.207; 95% CI: 1.745, 15.536; *P* = 0.003) (Figure 2B).

### 3.5 Comparison of liver function parameters between experimental and control groups

**FIGURE 3:**
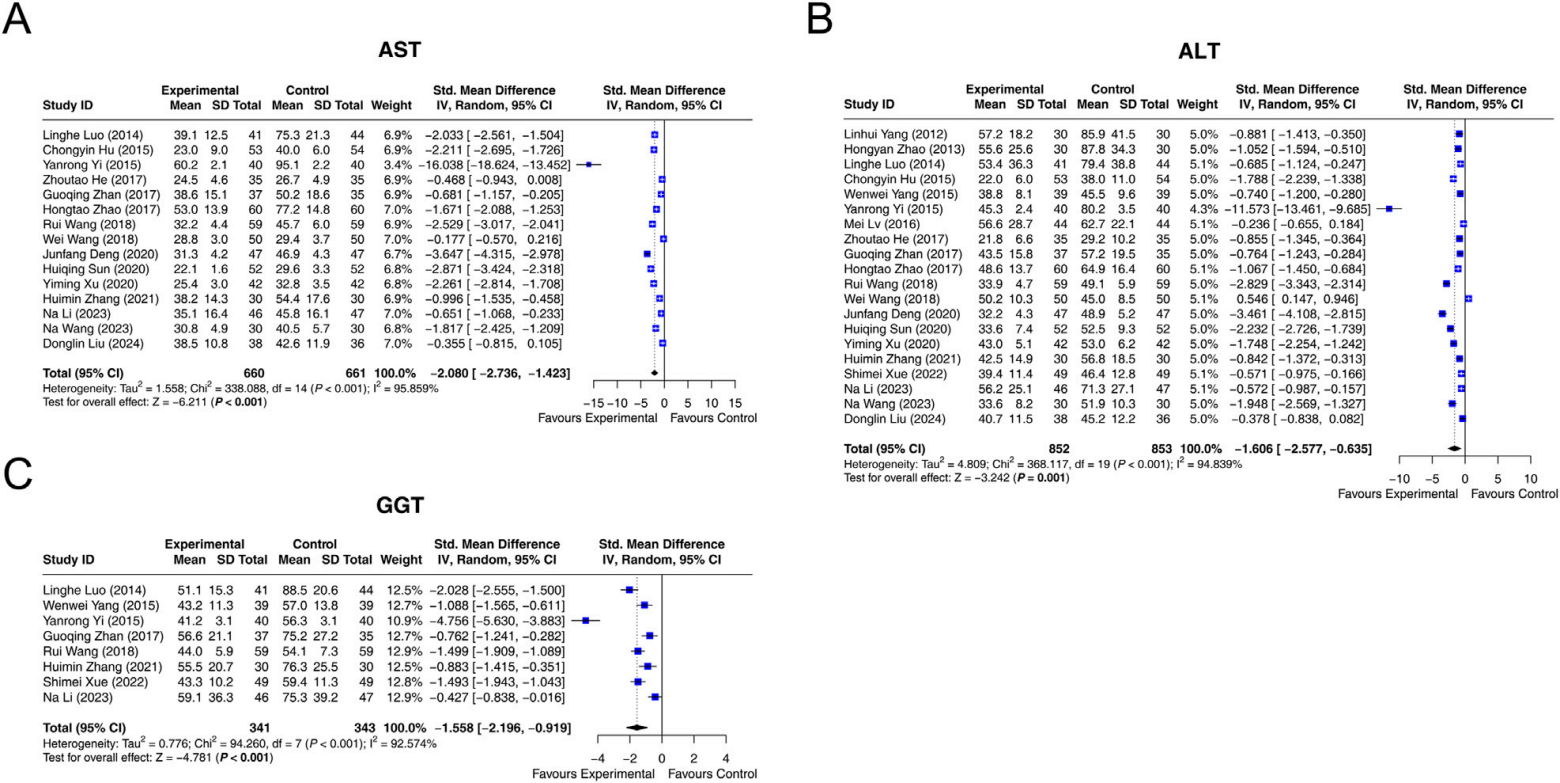
Forest plots of liver function parameters. AST **(A)**, ALT **(B)**, and GGT **(C)** in the control and experimental groups.

Fifteen studies reported that AST with heterogeneity existing among them (I^2^ = 95.859%, *P* < 0.001). The random-effects model indicated that, compared with interventions without LCBE enteric-coated capsules, interventions containing LCBE enteric-coated capsules reduced AST (SMD: −2.080; 95% CI: −2.736, −1.423, *P* < 0.001) (Figure 3A).

Data on ALT was extracted from 20 studies. Heterogeneity existed among these studies (I^2^ = 94.839%, *P* < 0.001). According to the random-effects model, compared with interventions without LCBE enteric-coated capsules, interventions containing LCBE enteric-coated capsules decreased ALT (SMD: −1.606; 95% CI: −2.577, −0.635, *P* = 0.001) (Figure 3B).

GGT was reported in 8 studies, and heterogeneity existed among them (I^2^ = 92.574%, *P* < 0.001). The random-effects model suggested that interventions containing LCBE enteric-coated capsules reduced GGT compared with interventions without LCBE enteric-coated capsules (SMD: −1.558; 95% CI: −2.196, −0.919, *P* < 0.001) (Figure 3C).

### 3.6 Comparison of metabolic parameters between experimental and control groups

**FIGURE 4:**
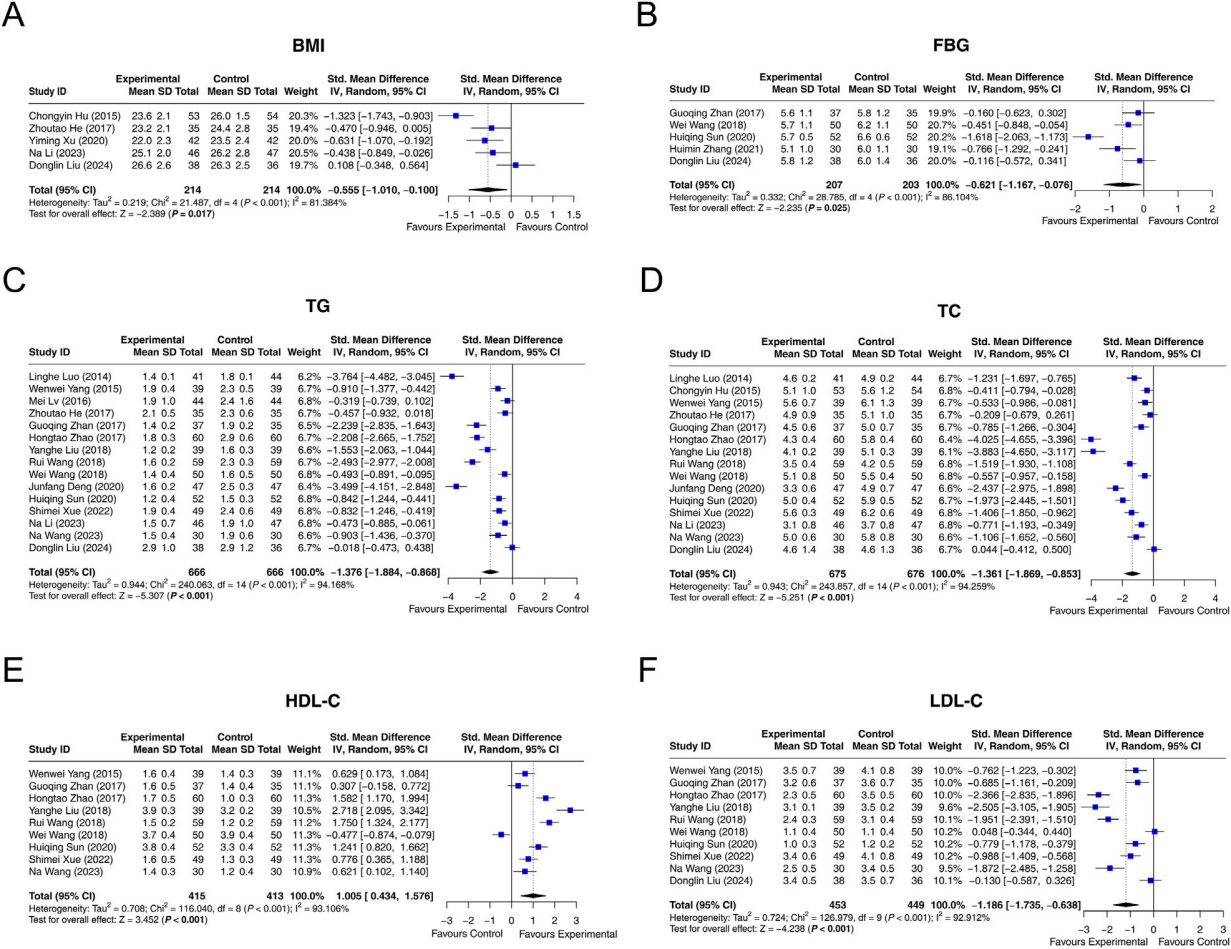
Forest plots of metabolic parameters. BMI **(A)**, FBG **(B)**, TG **(C)**, TC **(D)**, HDL-C **(E)**, and LDL-C **(F)** in the control and experimental groups.

Five studies reported data on BMI, and heterogeneity was observed among them (I^2^ = 81.384%, *P* < 0.001). The random-effects model revealed that interventions containing LCBE enteric-coated capsules reduced BMI compared with interventions without LCBE enteric-coated capsules (SMD: −0.555; 95% CI: −1.010, −0.100, *P* = 0.017) (Figure 4A).

Among 5 studies that reported FBG, heterogeneity was observed (I^2^ = 86.104%, *P* < 0.001). The random-effects model indicated that interventions containing LCBE enteric-coated capsules decreased FBG compared with interventions without LCBE enteric-coated capsules (SMD: −0.621; 95% CI: −1.167, −0.076, *P* = 0.025) (Figure 4B).

Fifteen studies reported TG. Heterogeneity was found among them (I^2^ = 94.168%, *P* < 0.001). According to the random-effects model, interventions containing LCBE enteric-coated capsules reduced TG compared with interventions without LCBE enteric-coated capsules (SMD: −1.376; 95% CI: −1.884, −0.868, *P* < 0.001) (Figure 4C).

Fifteen studies reported TC. Heterogeneity existed among them (I^2^ = 94.259%, *P* < 0.001). The random-effects model indicated that interventions containing LCBE enteric-coated capsules reduced TC compared with interventions without LCBE enteric-coated capsules (SMD: −1.361; 95% CI: −1.869, −0.853, *P* < 0.001) (Figure 4D).

HDL-C was reported in nine studies. There was heterogeneity among them (I^2^ = 93.106%, *P* < 0.001). The random-effects model disclosed that interventions containing LCBE enteric-coated capsules increased HDL-C compared with interventions without LCBE enteric-coated capsules (SMD: 1.005; 95% CI: 0.434, 1.576, *P* < 0.001) (Figure 4E).

Among 10 studies that reported LDL-C, heterogeneity was observed (I^2^ = 92.912%, *P* < 0.001). The random-effects model disclosed that interventions containing LCBE enteric-coated capsules decreased LDL-C compared with interventions without LCBE enteric-coated capsules (SMD: −1.186; 95% CI: −1.735, −0.638, *P* < 0.001) (Figure 4F).

### 3.7 Comparison of inflammatory markers between experimental and control groups

**FIGURE 5:**
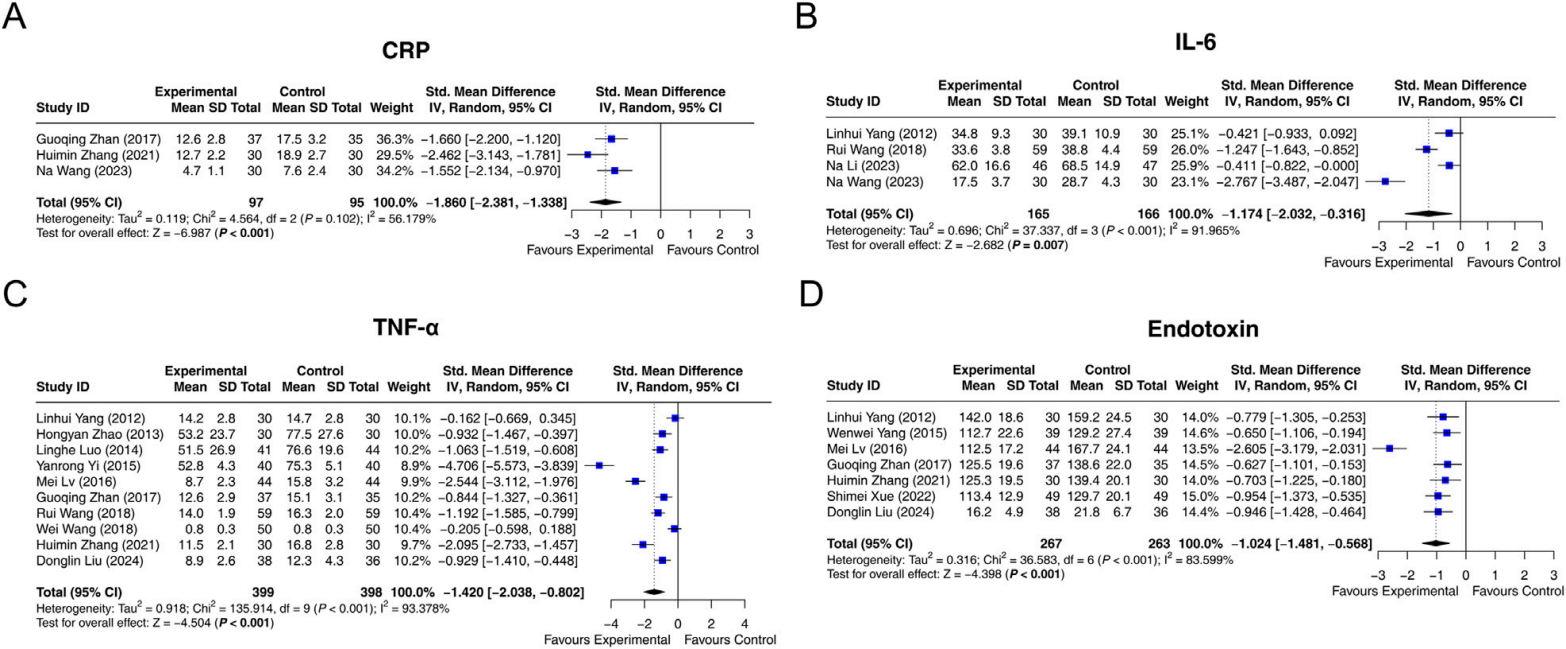
Forest plots of inflammation markers. CRP **(A)**, IL-6 **(B)**, TNF-α **(C)**, and endotoxin **(D)** in the control and experimental groups.

CRP was reported in 3 studies. Heterogeneity was found among these studies (I^2^ = 56.179%, *P* = 0.102). The random-effects model suggested that interventions containing LCBE enteric-coated capsules decreased CRP compared with interventions without LCBE enteric-coated capsules (SMD: −1.860; 95% CI: −2.381, −1.338, *P* < 0.001) (Figure 5A).

Four studies had data on IL-6. Heterogeneity was observed among these studies (I^2^ = 91.965%, *P* < 0.001). According to the random-effects model, interventions containing LCBE enteric-coated capsules reduced IL-6 compared with interventions without LCBE enteric-coated capsules (SMD: −1.174; 95% CI: −2.032, −0.316, *P* = 0.007) (Figure 5B).

Ten studies reported TNF-α, and heterogeneity was observed among them (I^2^ = 93.378%, *P* < 0.001). The random-effects model indicated that interventions containing LCBE enteric-coated capsules decreased TNF-α compared with interventions without LCBE enteric-coated capsules (SMD: −1.420; 95% CI: −2.038, −0.802, *P* < 0.001) (Figure 5C).

Endotoxin was reported in 7 studies. Heterogeneity existed among them (I^2^ = 83.599%, *P* < 0.001). The random-effects model disclosed that interventions containing LCBE enteric-coated capsules decreased endotoxin compared with interventions without LCBE enteric-coated capsules (SMD: −1.024; 95% CI: −1.481, −0.568, *P* < 0.001) (Figure 5D).

### 3.8 Publication bias and sensitivity analysis

Publication bias did not exist regarding most outcomes (all *P* > 0.05). However, publication bias existed regarding the outcomes of AST (*P* < 0.001), ALT (*P* < 0.001), TG (*P* = 0.004), and TC (*P* = 0.026) (Table 3).

**TABLE 3:** Publication bias.

Indicators	*P* value-Begg’s	Bias estimate (SE)	Adjust effect size [95% CI]^a^
Effective rate	0.099	11.000 (6.658)	(−)
Normal and light fatty liver rate	0.602	1.000 (1.915)	(−)
AST	**<0.001**	−67.000 (20.207)	−1.062 [−1.779, −0.346]
ALT	**<0.001**	−112.000 (30.822)	−1.148 [−2.534, 0.238]
GGT	0.458	−6.000 (8.083)	(−)
BMI	0.624	2.000 (4.083)	(−)
FBG	1.000	0.000 (4.083)	(−)
TG	**0.004**	−59.000 (20.207)	−1.200 [-1.738, −0.662]
TC	**0.026**	−45.000 (20.207)	−1.361 [-1.869, −0.853]
HDL-C	0.404	8.000 (9.592)	(−)
LDL-C	0.128	−17.000 (11.180)	(−)
CRP	0.602	−1.000 (1.915)	(−)
IL-6	0.497	−2.000 (2.944)	(−)
TNF-α	0.128	−17.000 (11.180)	(−)
Endotoxin	0.453	−5.000 (6.658)	(−)

SE, standard error; AST, aspartate transaminase; ALT, alanine aminotransferase; GGT, gamma-glutamyl transferase; TG, triglyceride; TC, total cholesterol; HDL-C, high-density lipoprotein cholesterol; LDL-C, low-density lipoprotein cholesterol; BMI, body mass index; FBG, fasting blood glucose; CRP, C-reactive protein; IL-6, interleukin-6; TNF-α, tumor necrosis factor-alpha.

^a^ Adjust effect size [95% CI] was the estimate after the trim-and-filling method if there was significant publication bias. Bold values indicated the statistically significant results.

Omitting any of the studies would not alter the outcomes of the effective rate, GGT, TG, TC, HDL-C, LDL-C, CRP, TNF-α, and endotoxin. However, omitting Na Li (2023) would affect the outcomes of normal and light fatty liver rate, omitting Yi and Zeng (2015) would affect the outcomes of AST, omitting filled Yi and Zeng (2015) would alter the outcomes of ALT, omitting Xu (2020) and Li et al. (2023) would affect the outcomes of BMI, omitting Wang et al. (2018) and Zhang (2021) would affect the outcomes of FBG, and omitting Wang (2018) would affect the outcomes of IL-6 (Supplementary Figures S1A–O).

## 4 Discussion

As a gut microbiota intervention, probiotics exhibit satisfactory efficacy in treating MAFLD patients, according to previous meta-analyses (Ma et al., 2013; Xiao et al., 2019). However, relevant evidence regarding LCBE enteric-coated capsules is scarce. This meta-analysis discovered that the effective rate and normal and light fatty liver rate in MAFLD patients receiving interventions containing LCBE enteric-coated capsules were 2.576 and 5.207 times, respectively, of those in patients receiving interventions without LCBE enteric-coated capsules. A potential reason might be that LCBE enteric-coated capsules could enhance liver function, improve metabolic status, reduce inflammation, restore the intestinal barrier, and modulate gut microbiota composition, thereby attenuating MAFLD progression (Jiang et al., 2021).

Probiotics possess the ability to improve liver function by regulating intestinal microbiota and systemic inflammation (Milosevic et al., 2019; Safari and Gerard, 2019; Di Vincenzo et al., 2024). Several meta-analyses have reported the effect of probiotics on liver function parameters in MAFLD patients (Ma et al., 2013; Musazadeh et al., 2022; Kanchanasurakit et al., 2022). For example, a previous meta-analysis indicated that probiotic therapies reduced ALT and AST in MAFLD patients (Ma et al., 2013). Another previous meta-analysis discovered that probiotics could lower ALT, AST, and GGT levels in MAFLD patients (Musazadeh et al., 2022). In line with these previous meta-analyses (Ma et al., 2013; Musazadeh et al., 2022), we also discovered that interventions containing LCBE enteric-coated capsules decreased AST, ALT, and GGT compared to those without LCBE enteric-coated capsules in MAFLD patients. Our findings suggested that interventions containing LCBE enteric-coated capsules could improve liver function in MAFLD patients.

The interplay between metabolic dysfunction and gut microbiota drives the progression of MAFLD (Tilg et al., 2021). Previous meta-analyses have disclosed the effect of probiotics on metabolic parameters in MAFLD patients (Kanchanasurakit et al., 2022; Liu et al., 2019; Kazeminasab et al., 2024). For instance, probiotics decreased TG compared to placebo in MAFLD patients (Kanchanasurakit et al., 2022). Another previous meta-analysis reported that probiotics plus exercise reduced LDL-C and TC compared to exercise alone in MAFLD patients (Kazeminasab et al., 2024). In accordance with these previous meta-analyses, we discovered that BMI, FBG, TG, TC, and LDL-C were reduced, and HDL-C was increased by interventions containing LCBE enteric-coated capsules compared to those without LCBE enteric-coated capsules in MAFLD patients. Our findings indicated that interventions containing LCBE enteric-coated capsules were beneficial in improving metabolic status in MAFLD patients.

Gut dysbiosis can facilitate the production of endotoxins, which further promotes the release of proinflammatory cytokines, thereby accelerating the progression of MAFLD (Buzzetti et al., 2016). Previous meta-analyses revealed that gut microbiota interventions could reduce inflammation, as evidenced by reduced TNF-α, IL-6, lipopolysaccharides, and CRP in MAFLD patients (Carpi et al., 2022; Pan et al., 2024; Pan et al., 2020). Consistent with the findings of these previous meta-analyses, we found that inflammatory markers, including CRP, IL-6, TNF-α, and endotoxin, were reduced by interventions containing LCBE enteric-coated capsules compared to those without LCBE enteric-coated capsules in MAFLD patients. As explained by a previous study, the effect of LCBE on lowering inflammation might be through the toll-like receptor 4/nuclear factor kappa-B pathway (Jiang et al., 2021). Considering that aggravated inflammation could promote MAFLD progression, LCBE enteric-coated capsules could be given prophylactically in patients with early-stage MAFLD, which might be beneficial in attenuating disease progression.

In this meta-analysis, several liver function parameters, metabolic parameters, and inflammation markers were extracted from the enrolled studies. Clinically, liver function parameters, including ALT, AST, and GGT, have greater importance in clinical practice due to their ability to directly reflect the degree of liver injury. The abnormal metabolic parameters or inflammatory markers may be relevant, but may not be directly related to liver injury. Therefore, ALT, AST, and GGT possess higher priority in the evaluation of MAFLD.

Gut dysbiosis plays a crucial role in the progression of MAFLD by inducing liver injury, increasing liver inflammation and fibrosis, and causing metabolic dysfunction (Leung et al., 2016). Without proper control, gut dysbiosis can accelerate the progression of MAFLD to cirrhosis and hepatocellular carcinoma (Fang et al., 2022). Therefore, regulating gut dysbiosis is a promising strategy to improve the management of MAFLD patients. This meta-analysis discovered that interventions containing LCBE enteric-coated capsules improved liver function and metabolic status, and reduced inflammation compared to interventions without LCBE enteric-coated capsules in MAFLD patients. In clinical practice, this probiotic may be recommended for the treatment of MAFLD. However, Enterococcal species, such as *Enterococcus faecalis* and *E. faecium*, exhibit pathogenic genes, potentially leading to severe illness, disability, and death (Boeder et al., 2024; Wei et al., 2024). Considering pathogenic genes of *E. faecium* may impact the treatment outcomes, careful monitoring is required for MAFLD patients receiving LCBE enteric-coated capsules.

Several limitations existed among the included studies. (Han et al., 2023). To better understand the relative advantages of LCBE enteric-coated capsules, the comparison of this regimen with other probiotics in MAFLD patients, such as *Lactobacillus* and *Bifidobacterium*, could be further investigated. (Teng et al., 2023). Liver fibrosis plays a crucial role in the progression of MAFLD. However, only two included studies reported liver fibrosis-related markers, which hindered us from conducting a pooled analysis. Therefore, further studies could consider exploring the effect of LCBE enteric-coated capsules on liver fibrosis by evaluating liver fibrosis-related markers, such as liver stiffness and fibrosis scores, in MAFLD patients. (Grander et al., 2023). The included studies only involved adult MAFLD patients. Therefore, the efficacy of interventions containing LCBE enteric-coated capsules in pediatric MAFLD patients should be further investigated. (Leow et al., 2023). According to the quality assessment, some studies were assessed as having unclear or high risks of bias, particularly in bias due to missing outcome data and bias in the measurement of the outcome. These factors might affect the precision of the findings of this meta-analysis. (Powell et al., 2021). Since no enrolled studies reported the results stratified by gender, the impact of gender on the efficacy of LCBE enteric-coated capsules in MAFLD patients was unclear. This aspect could be further explored.

In conclusion, interventions containing LCBE enteric-coated capsules show satisfactory efficacy, which can improve liver function, metabolic status, and inflammation compared to those without LCBE enteric-coated capsules in MAFLD patients. Among the evaluated parameters, ALT, AST, and GGT should be given priority in clinical practice due to their ability to directly reflect the degree of liver injury. Further studies could consider optimizing the study design, increasing the sample size, evaluating the long-term efficacy, and comparing LCBE enteric-coated capsules with other probiotics to better guide clinical practice.

## Data Availability

The original contributions presented in the study are included in the article/Supplementary Material, further inquiries can be directed to the corresponding author.
