# Supplementary material for: Efficacy of interventions with live combined Bacillus subtilis and Enterococcus faecium enteric-coated capsules in metabolic associated fatty liver disease patients: a meta-analysis of randomized controlled trials
Source: Front Pharmacol. 2025 May 27;16:1610426. doi: 10.3389/fphar.2025.1610426 (PMC12148908; doi:10.3389/fphar.2025.1610426)
Supplement: Supplementary file 1 [file Image1.pdf]

# *Supplementary Materials*

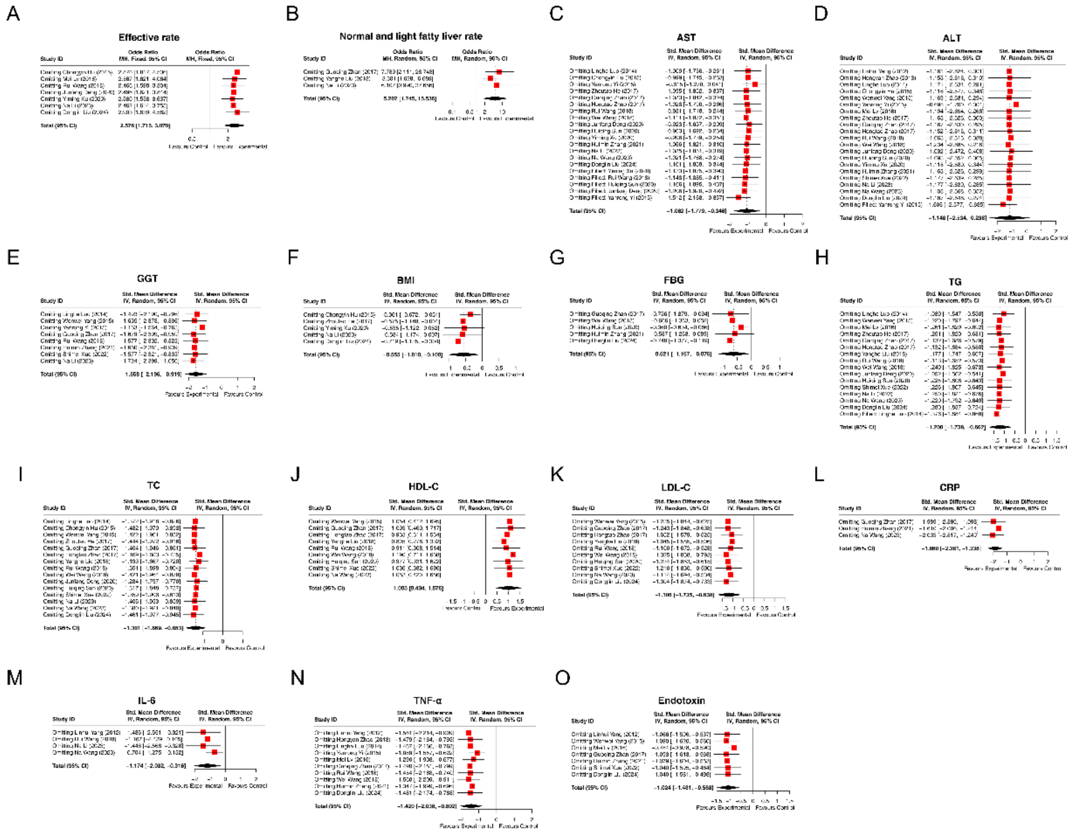


**Supplementary Figure 1.** Sensitivity analysis. Sensitivity analyses of effective rate (A), normal and light fatty liver rate (B), AST (C), ALT (D), GGT (E), BMI (F), FBG (G), TG (H), TC (I), HDL-C (J), LDL-C (K), CRP (L), IL-6 (M), TNF-α (N), and endotoxin (O).
